# Feasibility and Acceptability of *Fear-Less*: A Stepped-Care Program to Manage Fear of Cancer Recurrence in People with Metastatic Melanoma

**DOI:** 10.3390/jcm9092969

**Published:** 2020-09-14

**Authors:** Fiona A Lynch, Lynda Katona, Michael Jefford, Allan Ben Smith, Joanne Shaw, Haryana M Dhillon, Steve Ellen, Jo Phipps-Nelson, Julia Lai-Kwon, Donna Milne, Lahiru Russell, Victoria Dax, Justine Diggens, Holly Kent, Alison Button-Sloan, Jane Elliott, Mark Shackleton, Hayley Burridge, Maria Ftanou

**Affiliations:** 1 Psychosocial Oncology Program, Peter MacCallum Cancer Centre, Melbourne, VIC 3000, Australia; steve.ellen@petermac.org (S.E.); victoria.dax@petermac.org (V.D.); justine.diggens@petermac.org (J.D.); jane.rws@gmail.com (J.E.); maria.ftanou@petermac.org (M.F.); 2 Department of Psychology and Consultation Liaison Psychiatry, Alfred Health, Melbourne, VIC 3004, Australia; l.katona@alfred.org.au (L.K.); holly.kent@alfred.org.au (H.K.); 3 Department of Medical Oncology, Peter MacCallum Cancer Centre, Melbourne, VIC 3000, Australia; michael.jefford@petermac.org (M.J.); julia.lai-kwon@icr.ac.uk (J.L.-K.); 4 Australian Cancer Survivorship Centre, Melbourne, VIC 3000, Australia; 5 Sir Peter MacCallum Department of Oncology, Faculty of Medicine, Dentistry and Health Sciences, The University of Melbourne, Melbourne, VIC 3010, Australia; Jo.Phipps-Nelson@petermac.org; 6 Centre for Oncology Education and Research Translation, Ingham Institute for Applied Medical Research, South Western Sydney Clinical School, University of New South Wales, Kensington, NSW 2052, Australia; ben.smith@unsw.edu.au; 7 Psycho-Oncology Co-operative Research Group (PoCoG), School of Psychology, The University of Sydney, Sydney, NSW 2006, Australia; joanne.shaw@sydney.edu.au (J.S.); haryana.dhillon@sydney.edu.au (H.M.D.); 8 Centre for Medical Psychology & Evidence-based Decision-making, School of Psychology, The University of Sydney, Sydney, NSW 2006, Australia; 9 Department of Psychiatry, The University of Melbourne, Melbourne, VIC 3010, Australia; 10 Health Services and Implementation Science, Peter MacCallum Cancer Centre, Melbourne, VIC 3000, Australia; donna.milne@petermac.org; 11 Department of Skin and Melanoma Service, Peter MacCallum Cancer Centre, Melbourne, VIC 3000, Australia; 12 School of Nursing and Midwifery, Institute for Health Transformation, Deakin University, Geelong, VIC 3217, Australia; l.russell@deakin.edu.au; 13 Centre for Quality and Patient Safety—Eastern Health Partnership, Box Hill, VIC 3128, Australia; 14 Melanoma Patients Australia, Melbourne, VIC 3000, Australia; buttonsloan@bigpond.com; 15 Melanoma Research Victoria Consumer Reference Group, Melbourne, VIC 3000, Australia; 16 Australian Melanoma Consumer Alliance, Melbourne, VIC 3000, Australia; 17 Department of Medical Oncology, Alfred Health, Melbourne, VIC 3004, Australia; mark.shackleton@monash.edu (M.S.); h.burrdige@alfred.org.au (H.B.); 18 Department of Medicine, Monash University, Melbourne, VIC 3010, Australia; 19 Centre for Mental Health, Melbourne School of Population and Global Health, The University of Melbourne, Melbourne, VIC 3010, Australia

**Keywords:** fear of cancer recurrence, stepped care, metastatic melanoma

## Abstract

Immunotherapies and targeted therapies have revolutionised treatment of metastatic melanoma and improved survival rates. However, survivors treated with novel therapies are vulnerable to high levels of fear of cancer recurrence or progression (FCR). Existing FCR interventions have rarely been trialled in people with advanced cancer. The current study aimed to evaluate the acceptability and feasibility of *Fear-Less*: a stepped-care model to treat FCR in people with metastatic melanoma treated with immunotherapy or targeted therapy. Sixty-one outpatients with metastatic melanoma were screened using the Fear of Cancer Recurrence Inventory Short Form (FCRI-SF) and Fear of Progression Questionnaire Short Form (FoP-Q-SF). Survivors with subthreshold FCR were stratified to a self-management intervention while those with clinical levels of FCR were provided with an individual therapy, *Conquer Fear*. Survivor experience surveys and rescreening were administered post-intervention completion. Results indicated that *Fear-Less* was an acceptable and feasible FCR intervention. Results provided preliminary support for the potential impact of *Fear-Less* in reducing FCR. *Fear-Less* is a promising first step in providing an acceptable and feasible stepped-care model to treat FCR in survivors with metastatic disease.

## 1. Introduction

Immunotherapy and targeted therapy have revolutionised the management of metastatic melanoma and substantially improved survival for a subset of patients [1]. Historically, the median life expectancy for people with stage IV melanoma was seven months [2]. In contrast, the five-year overall survival rate for the combination of a B-Raf proto-oncogene (BRAF) and mitogen-activated protein kinase kinase (MEK) inhibitor (dabrafenib and trametinib) is now 37% [3]. Similar or better survival has been found for immunotherapies, with five-year overall survival of 38.7% for single-agent pembrolizumab [4], 44% for single-agent nivolumab [5], and 52% for combination ipilimumab and nivolumab [5]. Despite this, individuals endure considerable fear and uncertainty regarding the likelihood and durability of their response to treatment [6]. Regular monitoring and extended treatment duration may also place these survivors at high risk of fear of cancer recurrence or progression (FCR) [6].

FCR is defined as the fear, worry, or concern relating to the possibility that cancer will come back or progress [7]. A recent Australian survey found that up to 86% of survivors with metastatic melanoma report some degree of FCR [8]. FCR involves intrusive thoughts, worries, hypervigilance to physical symptoms and either avoidance of or excessive levels of symptom checking [7]. It is often heightened in the days or weeks before receiving scan results or attending medical reviews [9]. In people with early stage disease, clinical levels of FCR have been associated with poor day-to-day functioning, reduced quality of life and increased rates of depression, anxiety, and worry about the future [10]. Untreated FCR has also been associated with avoidance of, or excessive requests for, medical reviews, examinations or follow-up visits. This may result in adverse health outcomes for survivors and increase health costs to survivors and health services [10,11,12].

Over the past decade, several psychological interventions have been developed and trialled to address FCR in cancer survivors, primarily those with early stage disease. FCR interventions have been delivered in individual, group, and online formats. Survivors who have completed these interventions have experienced significant reductions in reported levels of FCR, depression, anxiety, stress and improved overall quality of life [11,13,14,15,16,17,18,19,20]. Of the established interventions, *Conquer Fear* has demonstrated efficacy in early stage disease, with post-intervention reductions in FCR maintained at 3 and 6 month follow up [14]. *Conquer Fear* is based on the Common-Sense Model (CSM) of illness, the Self-Regulatory Executive Function model (S-REF), and Relational Frame Theory (RFT) [21]. It is a five-session face-to-face intervention that aims to manage worry and excessive threat monitoring, address existential issues, and promote goal setting [22]. *Conquer Fear* has recently been adapted to a five-module online self-management intervention, iConquerFear [23]. iConquerFear is hoped to increase accessibility of FCR interventions, and is currently being trialled in people with early stage colorectal cancer [24]. Both *Conquer Fear* and iConquerFear remain untested in survivors with advanced cancer and their acceptability to them unknown.

To date, only one randomised controlled trial (RCT) has evaluated the efficacy of an FCR intervention that specifically targets FCR in survivors with advanced malignancies. This compared Cognitive Behaviour Group Therapy (CBT) and Supportive-Experiential Group Therapy (SET) in survivors in rehabilitation with either metastatic cancer or disease recurrence. These researchers found that both CBT and SET significantly reduced FCR, depression, and anxiety, and improved health-related quality of life [16]. However, the transferability of the findings is limited as the interventions were only delivered in group settings to rehabilitation participants likely to have high levels of motivation.

Despite the growing number of FCR interventions, very few cancer survivors have access to them. Barriers to access include lack of routine identification of FCR [25], poor referral rates, and limited availability of therapists with specialised training to deliver these intensive interventions [22]. Developing acceptable intervention models that enable FCR treatment to be embedded in routine care has been identified as the top FCR research priority [22].

A stepped-care FCR model could provide a solution to overcome these barriers and increase access to FCR interventions by offering different intensity interventions based on survivors’ level of need. This is an approach recommended in the Cancer Australia (the peak national government cancer agency) guidelines for managing FCR [26]. A low-intensity intervention targets mild or moderate symptoms, is readily accessible, convenient for survivors, and requires minimal specialist therapist time to deliver [27]. Examples include psychoeducation [28], brief low-intensity CBT [29], or guided self-management via online or booklet [30]. In contrast, a high-intensity intervention would address high levels of symptoms which usually involves greater clinician involvement such as face-to-face psychological therapy.

Developing a stepped-care approach to treat FCR in survivors with metastatic disease treated with novel therapies could provide tailored interventions that are accessible, cost effective, and relevant to this emerging survivor population.

To better manage FCR in survivors with metastatic melanoma treated with novel therapies, we developed a stepped-care model (*Fear-Less*). Survivors with subthreshold levels of FCR were offered a self-management intervention and survivors with clinical levels of FCR were offered individual therapy based on the *Conquer Fear* protocol. The primary aim of this study was to evaluate the feasibility and acceptability of *Fear-Less*. A secondary aim was to conduct preliminary evaluation of the impact of the stepped-care model on FCR outcomes.

## 2. Materials and Methods

### 2.1. Participants

Participants were recruited by research staff at melanoma outpatient appointments at two metropolitan hospitals in Melbourne, Australia. Patients were eligible if they were 18 years or older, had a histologically confirmed diagnosis of stage IV metastatic melanoma, were at least six months post-initiation of immunotherapy and/or targeted therapy, had a complete partial or stable response on their most recent imaging, and could read or write in English. The research team attempted to approach all eligible patients attending outpatient appointments on days of screening unless their treating team advised they were too unwell (*n* = 17). The target sample for screening was 60 participants. This study was approved by the governing hospital’s Human Research Ethics Committee (HREC reference number LNR/47175/PMCC-2018).

### 2.2. Study Measures

#### 2.2.1. Demographics and Medical History

Demographic information and relevant medical history were extracted from the medical record including age, gender, disease status and treatment history.

#### 2.2.2. FCR Assessments

There is limited data on the use of FCR measures in people with advanced cancer. The combination of the below measures was therefore used to ensure that FCR was adequately captured.

- Fear of Cancer Recurrence Inventory Short Form (FCRI-SF; [31]). The FCRI-SF is a 9-item self-report questionnaire that was used as a screening and outcome measure to assess FCR. The questionnaire assesses the presence, frequency, intensity, and duration of thoughts associated with FCR. Total scores range from 0 to 36, with higher scores indicating higher levels of FCR. A score of 13–21 was used to identify participants with subthreshold FCR [31,32], while a score of ≥22 was used to identify participants with high levels of FCR [32,33]. The FCRI-SF has shown strong internal consistency (alpha = 0.95), temporal stability (*r* = 0.89), and construct validity [31].
- Fear of Progression Questionnaire Short Form (FoP-Q-SF; [34]). The FoP-Q-SF is a 12-item self-report questionnaire used as a screening and outcome measure to assess the level of fear of cancer progression. Total scores range from 12 to 60. A cut-off score of 24–33 was used to suggest subthreshold levels of fear of progression [35] and a score ≥34 was used to identify participants with high levels of fear of progression [36]. FoP-Q-SF has shown high reliability (Cronbach’s alpha = 0.87 to 0.90), with evidence of construct validity [34,37].

Participants whose scores reached different thresholds according to the two measures were offered the intervention corresponding with their highest score. For example, a participant who scored in the subthreshold range on one measure and the clinical FCR range on the other measure would be offered the individual therapy pathway.

#### 2.2.3. Acceptability and Feasibility

To assess acceptability and feasibility of screening and interventions, three purposely designed survivor experience surveys (see Supplementary Material) were administered:

- The Screening Survivor Experiences Survey was administered to all participants directly after screening. This seven-item measure included rating scales of the degree to which they agreed or disagreed that the screening questionnaires were easy to complete, easy to understand, and acceptable. Open-response items asked participants about how important they considered screening for FCR.
- The Self-Management Survivor Experiences Survey was administered after completion of the self-management intervention. The 13-item measure assessed participants’ previous experience of FCR support, the most and least useful aspects of the intervention, whether they would recommend the intervention and subjective changes in FCR.
- The Individual Therapy Survivor Experiences Survey was administered after the individual therapy. This 10-item measure assessed the participants’ previous FCR supports, experience of the individual therapy, whether they would recommend it, and subjective changes in FCR.

#### 2.2.4. Operational Data

Clinician time taken to deliver screening and interventions, uptake and adherence rates, follow up, and reasons for declining were collected.

### 2.3. Study Procedures

Over a 16 week period from February 2019 to June 2019, eligible survivors were screened for FCR using the FCRI-SF and FoP-Q-SF by a psychology researcher or clinical psychologist. Participants with low levels of FCR did not require FCR intervention and received treatment as usual: standard care from their primary care medical and nursing staff. Participants identified as having subthreshold levels of FCR were offered the self-management intervention and participants who were identified as having clinical FCR were offered individual therapy.

At the completion of the self-management intervention or individual therapy, participants were provided with rescreening questionnaires and a survivor experiences survey.

#### 2.3.1. Stepped Care Intervention

**Figure 1:**
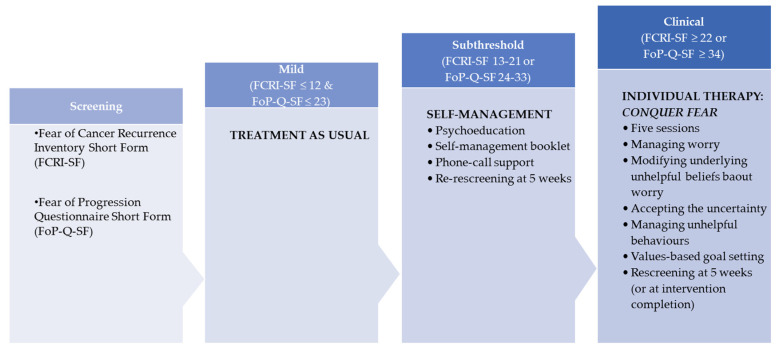
Stepped-care interventions.

The three levels of interventions are described below and summarised in Figure 1.

##### Step 1: Treatment as Usual

No further follow up or additional screening was undertaken. Participants continued with their usual care.

##### Step 2: Self-Management Intervention

Participants who were identified as having subthreshold levels of FCR based on screening or clinical assessment were offered the self-management intervention. Participants received a copy of the purpose-designed self-management booklet. To develop the self-management booklet, the following steps were taken: (1) a scoping review of the literature to collate and review relevant national and international resources, (2) focus groups or interviews with melanoma survivors and content experts to inform content and design, and (3) a quality review process to ensure relevance and utility of the booklet.

The self-management booklet included psychoeducational material and strategies on key cognitive behavioural skills to manage FCR, healthy living, and information about how to cope with disease progression. The booklet included seven activities to deepen participants’ understanding of their FCR and build their coping skills. This included (1) identifying values, (2) setting goals, (3) relaxation, (4) identifying triggers, (5) identifying unhelpful thoughts, (6) focusing on things within one’s control, and a (7) FCR management plan. Acceptable intervention completion levels were defined as self-reported reading 75% or more of the self-management booklet.

Upon receiving the booklet participants were oriented to its use and encouraged to read the sections that appeared most relevant to their experience. Three weeks after receiving the booklet, a phone call was made to all participants by the psychology researcher or clinical psychologist to reinforce new skills acquired and problem solve any difficulties experienced with using the booklet.

##### Step 3. Individual Therapy

Participants identified as having high levels of FCR on screening underwent a clinical assessment to confirm severity of FCR and need for individual therapy. Participants whose assessment indicated clinical levels of FCR (high severity, frequency, intensity and impact of FCR) were offered the five individual sessions based on the *Conquer Fear* protocol. Sessions were delivered by a clinical psychologist trained in *Conquer Fear* and ranged from 60 to 90 min in duration. Each session was accompanied by home-based practice of skills learned in session and home reading to consolidate skill acquisition. Session content is outlined below:

- Session 1: FCR-specific assessment and model to explain treatment, discussion of existential changes brought on by cancer, value identification and goal setting.
- Session 2: Discussion of the impact of vulnerability factors on FCR; rationale and practice of the Attention Training Technique.
- Session 3: Introduction to detached mindfulness.
- Session 4: Discussion of self-examination practices and medical surveillance, discussion of metacognitive beliefs that underpin FCR, and worry postponement.
- Session 5: Review of goal-setting task, consolidation of skills, and relapse prevention planning.

Session 4 of the protocol was adapted to improve relevance to survivors with advanced disease on novel therapies by removing the generic recommendations usually provided on symptom monitoring. Acceptable level of the individual therapy was defined as completing four or more sessions.

### 2.4. Data Analysis

Descriptive statistics were used to summarise demographic, clinical, and operational data.

Qualitative responses of participants’ experiences were summarised using content analysis to assess acceptability of screening and interventions.

Feasibility of the stepped-care approach was assessed using percentage of participants approached who completed screening, percentage of participants who accepted the interventions, clinician time to deliver screening and interventions. Acceptability of the stepped-care approach was assessed using percentage adherence to the interventions, and qualitative analysis of survivor experience surveys.

Change scores between pre- and post-intervention FCRI-SF and FoP-Q-SF were calculated to describe the impact of the *Fear-Less* interventions. Participants whose post-intervention scores on the FCRI-SF or FoP-Q-SF decreased by 10% or more (indicative of clinically significant improvement [38]) were considered to have a reduction in FCR.

## 3. Results

### 3.1. Stepped-Care Pathways

**Figure 2:**
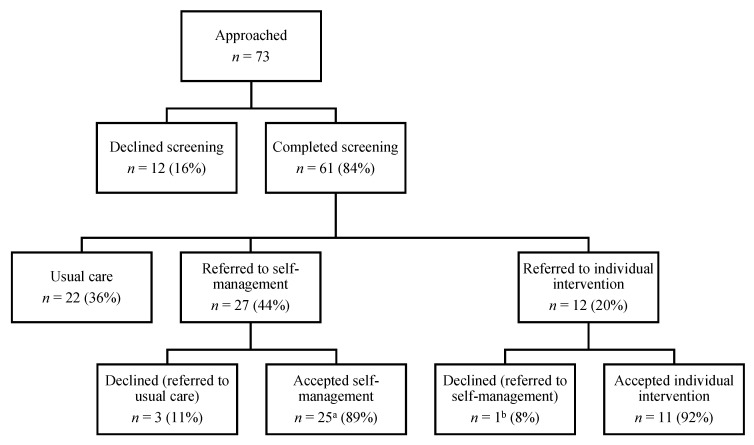
Stepped-care pathways of *Fear-Less*. ^a^ Includes one survivor who declined individual therapy and accepted self-management. ^b^ Referred to self-management instead.

Seventy-three people were approached for FCR screening and 61/73 (84%) completed screening (Figure 2). Seven (10%) declined screening due to time commitments and five (7%) did not return screening questionnaires after consenting. Thirty-nine participants (64%) were identified as experiencing FCR. Twenty-seven (44%) experienced subthreshold levels of FCR and were eligible for the self-management intervention, and 12 (20%) had clinical levels of FCR requiring individual therapy.

Ninety percent of those referred to *Fear-Less* interventions accepted the referral, including 24/27 (89%) who accepted the self-management intervention and 11/12 (92%) who accepted individual therapy. One participant who declined individual therapy accepted the self-management intervention.

Key socio-demographic, clinical and treatment characteristics of participants are shown in Table 1. The sample was primarily male (41, 67%), on active treatment (37, 61%) and treated with immunotherapies (43, 70%). The mean age was 61.39 years (SD = 11.62).

### 3.2. Feasibility and Acceptability

#### 3.2.1. Screening

All participants (61, 100%) who completed the screening measures completed the Screening Survivor Experiences Survey. The majority agreed or strongly agreed that the screening questionnaires were easy to complete (98%), easy to understand (98%), and time to complete screening questionnaires was acceptable (100%). Clinician time taken to administer the screening questionnaires ranged from one to 12 min (mean 3.25, SD = 2.30).

Screening identified 16 survivors with high levels of FCR but, of these, 12/16 (75%) were confirmed to have clinical levels of FCR at the clinical assessment. The remaining four had moderate levels of FCR and were referred to the lower intensity self-management intervention.

Qualitative responses on survivor experience surveys consistently described that screening for FCR was “very important”. Participants indicated screening validated their emotional experience and assisted with treatment planning. A small number of participants viewed the screening itself as therapeutic. The quotes below provide examples of these responses:

Participant 48: “I think it is very important and should be included as part of your treatment plan when first diagnosed and it can then be modified if and when required”.

Participant 22: “The questionnaire raised issues that I wasn’t aware I was fearful of and by identifying the issues helps me then to deal with them”.

Participant 50: “Filling in the questionnaire was thought provoking in itself, and a type of therapy”.

#### 3.2.2. Self-Management Intervention

**Figure 3:**
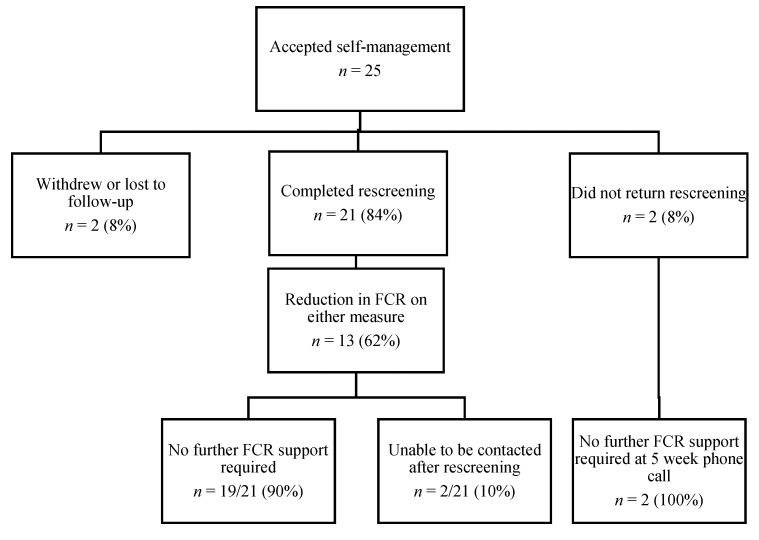
Self-management intervention flow of participants.

Twenty-seven participants were offered the self-management intervention after screening and 24 (89%) accepted the intervention. One additional participant accepted self-management after declining individual therapy, resulting in 25 participants commencing the self-management intervention. Three participants declined the self-management intervention as they felt that they were coping with their FCR. Figure 3 shows the flow of participants in the self-management intervention. Twenty-one participants (84%) completed the intervention and rescreening. On average, the self-management intervention took 20.62 min (SD = 9.77) of clinician time to deliver.

The majority of participants (13/21, 62%) read ≥50% of the booklet with 10 participants (48%) reading ≥75% of the booklet. Thirteen participants (62%) reported that they would recommend the self-management intervention to others. One-third of participants (7/21, 33%) were unsure whether they would recommend it with some preferring a face-to-face or online delivery and others reporting they believed they did not need the intervention. One participant indicated they would not recommend the intervention. However, their qualitative data had no indication of reasons they would not recommend it (e.g., “felt good about” participating; that it was helpful to see that “I’m not the only one in this boat”). In total, 33% (7/21) reported subjective improvements in FCR after the intervention.

Eight participants (38%) completed ≥3/7 activities and seven (33%) did not complete any. Participants identified that the most helpful activities were keeping a relaxation diary (7/21, 33%), goal setting (6/21, 29%) and identifying what was within their control (6/21, 29%).

When asked to describe the most useful aspects of the intervention, participants reported that the intervention was “interesting”, “easy to understand”, “helpful”, and “cover[ed] all aspects of living with cancer”. Participants also indicated that the self-management booklet increased awareness of FCR, had the potential to prevent FCR, and was a useful resource for family and friends. These sentiments are captured by the following statements:

Participant 10: “I have to say the book speaks for itself, it’s very easy to understand and put into practice, and helpful to putting strategies in place”.

Participant 41: “I think the book made me aware of things that I never thought about”.

Participants were also asked to describe the least useful aspects of the intervention. Three participants were concerned some sections of the self-management booklet could trigger anxiety or lead to worry about disease progression. The three participants (14%) who felt they did not need the intervention could not relate to words used in the booklet such as fear, care and intervention. An additional two participants (10%) described the booklet as difficult to understand or read.

#### 3.2.3. Individual Therapy

**Figure 4:**
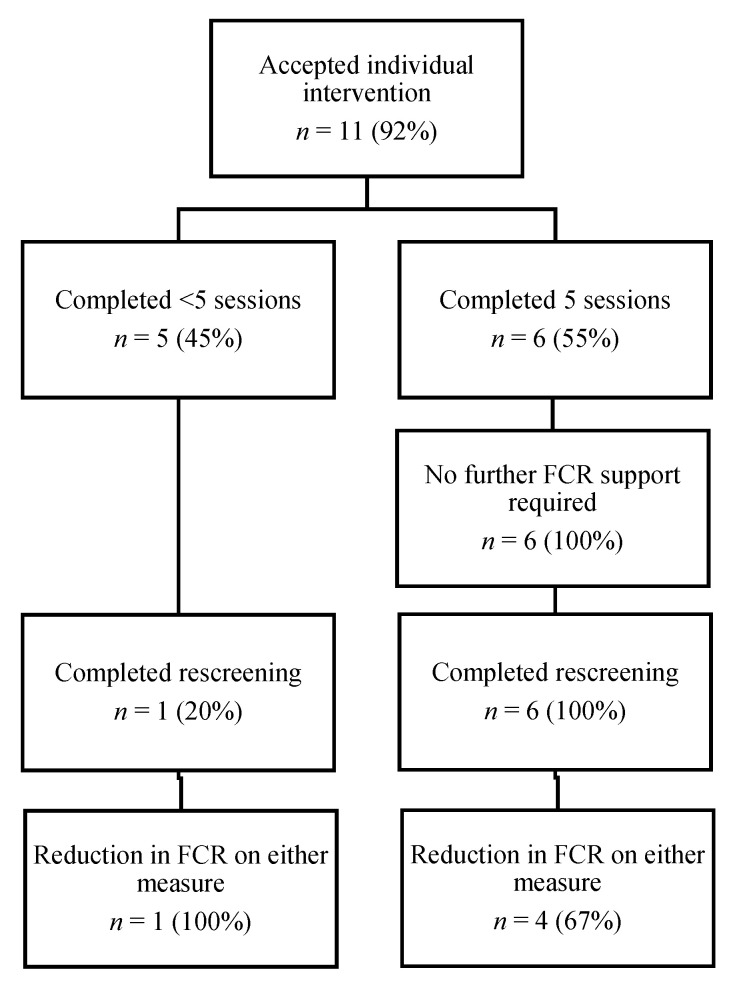
Individual therapy flow of participants.

A total of 12/61 participants (20%) were referred for individual therapy and 92% (11/12) accepted the referral. One declined due to the intense nature of the intervention but accepted a referral to the self-management intervention. Six participants (55%) attended all five sessions of the intervention, while two participants (18%) attended three sessions; two (18%) attended two sessions and one (9%) attended a single session. Reasons for completing <5 sessions included other significant psychosocial or health stressors taking priority over therapy (*n* = 3), returning to pre-existing community psychologist (*n* = 1), disengaged and then lost to follow up (*n* = 1). Figure 4 shows the flow of participants through the individual therapy.

Of the 11 participants who commenced individual therapy, seven (64%) completed post-intervention rescreening and survivor experience surveys. All six participants who completed five sessions reported that they would recommend the individual therapy to others. Participants’ qualitative responses identified that the most useful aspects of individual therapy were detached mindfulness (4/7) and being listened to and validated (3/7). All six participants who completed the intervention reported subjective improvements in FCR levels and all attributed this change to therapy. Some experiences are presented below:

Participant 11: “Practical tools provided are really useful. Also having the opportunity to try them during my support session”.

Participant 20: “The entire program was beneficial”.

Participant 28: “(Psychologist) made me feel comfortable and safe from the very beginning. I felt I could talk about my fear and other emotions freely”.

In terms of difficulties experienced with the individual therapy, two participants found discussions about past experiences confronting. One stated:

Participant 28: “In the beginning, I was asked to describe myself and my diagnosis. This brought about a lot of painful memories and brought to the forefront very negative feelings about my family that I felt I experienced throughout my cancer journey. I did not enjoy this, nor felt it was helpful”.

Participants were asked for recommendations on how the individual therapy could be improved. One participant recommended that oncologists ask about FCR and make direct referrals, one recommended greater clarity regarding program expectations, while another recommended including a peer support component.

Participant 20: “The only improvement would be for oncologists to suggest a program like this or suggest seeking assistance as many, like me, feel they are handling their diagnosis well, but in reality are struggling”.

### 3.3. Impact

#### 3.3.1. Self-Management Intervention

Post-intervention rescreening indicated that 62% of participants (13/21) had a reduction in FCR post-intervention compared to pre-intervention. After five weeks of self-management, all 21 participants were offered additional supports to manage FCR. However, 90% reported that they did not require any further support (two participants were uncontactable). Table 2 summarises the pre- and post-intervention FCRI-SF and FoP-Q-SF scores. The survivor experiences survey indicated that 17/21 (81%) self-management participants had never previously accessed professional help for FCR and 11/21 (52%) had never been asked about FCR.

Of the ten participants who read ≥75% of the booklet, 8/10 showed a reduction in FCR. The two participants who did not show any improvement attributed the lack of change in FCR to life stressors (e.g., a family member dying of or being diagnosed with terminal cancer prior to rescreening).

#### 3.3.2. Individual Therapy

Of the seven individual therapy participants who completed post-intervention evaluation and rescreening, six had never previously accessed professional help for FCR nor had they ever been asked about FCR. At the completion of individual therapy, 5/7 participants had a reduction in their FCR compared to pre-intervention. No participants who completed five sessions required further support for their FCR.

Two participants did not show improvements on rescreening. One attributed their lack of change on outcome measures to an increased frequency of thinking about FCR during the intervention without an increase in distress. The second participant attributed their lack of change to the complex nature of their illness.

## 4. Discussion

*Fear-Less* is a novel stepped-care model to treat FCR and the first known initiative designed to address FCR specifically in survivors with metastatic melanoma treated with novel therapies. *Fear-Less* appears to be a feasible and acceptable model of care with a high uptake of FCR screening (84%), self-management intervention (89%), and individual therapy (92%).

The majority of participants (61%) who required FCR intervention had never previously been asked about FCR and 82% had never had FCR treatment. This is particularly concerning given the long-term implications of FCR on quality of life and mental health [11,13,14,15,16,17,18,19,20]. Although it has been widely recommended by Butow et al. [39] that survivors are routinely screened for FCR, our results suggest FCR screening is not routinely implemented. Our study also found that participants want to discuss FCR early in treatment with their medical team. Barriers to FCR screening may include minimal health care professional knowledge or training in FCR [40], uncertainty regarding the optimal time across the cancer trajectory to assess FCR [23], and lack of access to FCR screening [25]. Our study supports the implementation capacity of routine FCR screening. As screening was self-reported, it required minimal instructions from clinicians, took approximately 3 min to administer, and survivors reported it was easy to complete, indicating that it can be integrated and administered in busy outpatient clinics with support of nursing or oncology staff. Brief training for health care professionals may be needed to improve confidence in identifying and addressing FCR [40].

Given the growing number of cancer survivors, self-management psychological interventions are becoming a popular part of cancer care. Our self-management intervention was acceptable and feasible to survivors with advanced disease, taking only 21 min of clinician time to support survivors through the intervention. Similar results were found in an Australian study that assessed the usability of iConquerFear with people with early stage cancer [23]. Smith et al. [23] found participants liked the flexibility of access of iConquerFear and the self-management intervention empowered participants to help themselves. However, iConquerFear is a fully self-directed resource, and some participants noted that they would benefit from having some clinician contact during the program. Another fully self-directed online CBT intervention to manage FCR, “Less fear after cancer”, had no effect on reducing FCR, leading the authors to recommend adding professional support to self-management interventions [41]. In contrast, an FCR intervention for survivors with early stage melanoma that included a psychoeducational resource and three telephone psychotherapeutic sessions was found to be effective in reducing FCR [15]. The acceptability and efficacy of self-management interventions may therefore be enhanced when including clinician support, which was used in the current self-management intervention.

Our findings provide preliminary evidence that the self-management intervention may have helped reduce FCR in this sample. Delivering a low-intensity intervention to those with subthreshold FCR may be just as effective as providing these survivors with a high-intensity intervention. For example, a recent study showed that survivors with low levels of FCR benefited equally from a low-intensity relaxation intervention compared to the high-intensity *Conquer Fear* [42]. This supports the stepped-care approach without compromising survivor outcomes.

Testing the cost utility of our intervention was beyond the scope of the project and the other self-management interventions for FCR also lacked an economic evaluation. However, other researchers have assessed costs of self-management psychological interventions in oncology and compared them to face-to-face interventions. For example, online CBT effective in reducing menopausal symptoms in breast cancer survivors was more cost effective than face-to-face group CBT for this cohort [43]. A self-management FCR intervention such as that developed in the current study may have the potential to provide an accessible effective treatment to those with subthreshold FCR with cost savings to the health care system.

Our study is the first to evaluate *Conquer Fear* in survivors with advanced disease and provides preliminary support that it may be an acceptable intervention for this population. All six participants who completed the five-session intervention would recommend it to others. The finding that one participant experienced increased frequency of FCR without increased distress may reflect a reduction in their previous unhelpful avoidant coping strategy. Avoidant coping is reported in individuals with higher levels of FCR [44] and reduced avoidance could indicate clinical improvement in overall FCR. Unlike the self-management intervention, *Conquer Fear* is highly resource dependent as it requires a minimum of nearly six hours of specialty therapist’s time to deliver. The individual therapy appeared to have an impact on reducing FCR for 5/7 of survivors who completed rescreening. Effective management of FCR in survivors with advanced disease is consistent with the study by Herschbach et al. [16] who found that CBT and SET were effective in reducing FCR in this population. Additional FCR factors not included in *Conquer Fear* may be particularly relevant to advanced disease (e.g., death anxiety) [45]. Future research could explore whether including these factors further increases the relevance and impact of FCR interventions in this population.

### 4.1. Clinical Implications

FCR in survivors with advanced melanoma treated with novel therapies largely goes undetected and untreated. Until further evidence amasses, it is recommended that the best evidence to date be used to screen and treat FCR in this population with great unmet need. Screening for FCR is essential and provides an opportunity for early intervention. There are several measures that could be used to screen for FCR [46]. Both the FCRI-SF and FoP-Q-SF have been used widely and have been evaluated with a number of different populations. However, further research is needed to determine the optimal FCR screening measure and cut offs in this population. Until clearer cut offs have been established, screening for both subthreshold and clinical levels of FCR could be complemented with a brief clinical assessment of the severity and frequency of FCR. Medical and nursing teams might be in the best position to initiate the conversation but may benefit from further training to increase confidence in FCR identification and management.

FCR self-management interventions with clinician involvement may help survivors work through the intervention and also help adjust the intervention to meet the individual needs of each survivor. Psycho-oncology nurses who are experts in supportive care, who know the survivors well and support survivors through their treatment regime could be the primary clinician supporting survivors through the self-management intervention and could refer to psycho-oncology services if further support is required.

*Conquer Fear* is efficacious in early stage disease with durable long-term outcomes. We demonstrated that it may also be suitable for survivors with stage IV melanoma treated with immunotherapies or targeted therapies. When delivering FCR interventions such as *Conquer Fear* to people with advanced disease on novel therapies, psycho-oncology experts may need to address specific fears related to treatment side-effects, uncertainty of symptom monitoring, and death anxiety.

### 4.2. Further Research

*Fear-Less* appears to be an acceptable and feasible model of care, and further evaluation of the efficacy and cost effectiveness of the *Fear-Less* stepped-care model within an RCT is needed. Further research is needed to explore how to better address FCR in survivors with other cancer types (e.g., lung cancer) who are treated with immunotherapies and targeted therapies. Very few FCR interventions address the needs of people who speak a language other than English, future research could focus on translating resources in other common languages (e.g., Arabic, Mandarin, Italian) and assess their usability.

### 4.3. Study Strengths and Limitations

This study had a number of strengths. Firstly, unlike previous interventions, *Fear-Less* is a stepped-care FCR intervention that is specifically tailored to survivors with advanced cancer. *Fear-Less* was also evaluated with a sample of both males and females, from a broad age range, across two sites. The use of two screening measures to assess FCR and fear of progression ensured that screening was adapted to this population with advanced disease. *Fear-Less* also targets FCR in survivors who have been treated with immuno- or targeted therapies, who may be at high risk of FCR.

This study also had several limitations. There is a lack of specificity in the screening measures and clinically significant cut-off scores [30]. Though individuals with high levels of FCR at screening completed a clinical assessment to confirm FCR severity, this was not provided to those with subthreshold FCR. This may have resulted in the self-management intervention being offered to participants who did not require any FCR intervention, as evidenced by qualitative feedback from three participants and a further three who declined the intervention. While the exploration of impact provides a preliminary understanding of the potential of these interventions, the small sample size and lack of a control group prevents conclusions being drawn about intervention efficacy. Our sample were all English-speaking individuals and therefore the suitability of this intervention for people from diverse backgrounds is unknown.

## 5. Conclusions

Stepped-care models such as *Fear-Less* have the potential to address FCR in survivors with advanced disease. Stepped-care models allocate the intensity of interventions based on survivors’ clinical level of need, and therefore increase access to interventions for the large number of survivors with untreated FCR. Survivors with metastatic melanoma want screening and treatment for FCR but most are not currently receiving it. Until further evidence amasses, FCR interventions for people with advanced disease treated with novel therapies should be carefully planned. Clinicians delivering these intervention should work closely with survivors to ensure that their needs are addressed.

## Figures and Tables

**Table 1:** Participants’ socio-demographic and treatment characteristics.

	FCR Screening Subcategories N = 61			Total Sample
	No FCR	Subthreshold FCR	Clinical FCR	
***n*** (%)	22 (36%)	27 (44%)	12 (20%)	**61**
**Age**				
Mean (SD)	69.2 (9.6)	60.0 (10.7)	50.3 (5.4)	61.4 (11.6)
Minimum	47	36	39	36
Maximum	82	76	60	82
**Sex**				
Male (%)	16(73%)	19 (70%)	6 (50%)	41 (67%)
Female (%)	6 (27%)	8 (30%)	6 (50%)	20 (33%)
**Treatment Agent Currently or Previously Received**				
Immunotherapies (%)	18 (82%)	19 (70%)	6 (50%)	43 (70%)
Targeted therapies (%)	2 (9%)	6 (22%)	3 (25%)	11 (18%)
Both immunotherapies and targeted therapies (%)	2 (9%)	2 (7%)	3 (25%)	7 (11%)
**Treatment Status**				
Having active treatment (%)	14 (64%)	14 (52%)	9 (75%)	37 (61%)
Treatment stopped ^a^ (%)	8 (36%)	13 (48%)	3 (25%)	24 (39%)
**FCRI-SF**				
Mean (SD)	6.95 (3.12)	17.22 (5.37)	23.42 (4.93)	14.74 (7.77)
Range	0, 11	6, 33	12, 31	0, 33
**FoP-Q-SF**	16.82 (3.79)	26.37 (6.41)	38.92 (9.26)	
Mean (SD)				25.39 (10.12)
Range	12–23	15–43	20–54	12–54

Note. *n* = Number. SD = standard deviation. FCRI-SF = Fear of Cancer Recurrence Inventory Short Form. FoP-Q-SF = Fear of Progression Questionnaire Short Form. FCR = Fear of Cancer Recurrence. ^a^ Due to completion of two years of therapy or toxicity.

**Table 2:** Stepped-care pre- and post-intervention scores on FCRI-SF and FoP-Q-SF.

	Self-Management	Individual Therapy
*n*	21	7
**FCRI-SF**		
Pre-intervention mean (SD)	17.67 (6.03)	24.29 (4.19)
Post-intervention mean (SD)	16.90 (7.69)	20.57 (6.35)
**FoP-Q-SF**		
Pre-intervention mean (SD)	29.14 (8.21)	37.29 (8.56)
Post-intervention mean (SD)	29.00 (7.79)	33.71 (7.87)

Note. SD = standard deviation. *n* = number. FCRI-SF = Fear of Cancer Recurrence Inventory Short Form. FoP-Q-SF = Fear of Progression Questionnaire Short Form.

## Supplementary Materials

The following are available online at https://www.mdpi.com/2077-0383/9/9/2969/s1.
