# Supplementary material for: Feasibility and Acceptability of Fear-Less: A Stepped-Care Program to Manage Fear of Cancer Recurrence in People with Metastatic Melanoma
Source: J Clin Med. 2020 Sep 14;9(9):2969. doi: 10.3390/jcm9092969 (PMC7565154; doi:10.3390/jcm9092969)
Supplement: Supplementary file 1 [file jcm-09-02969-s001.pdf]

THE PATIENT EXPERIENCE SURVEY

FEAR-LESS SCREENING QUESTIONNAIRES

Thank you for agreeing to participate in this survey about the *Fear-Less* Program. This survey focuses on your experiences of completing the program's screening questionnaires.

The survey may take up to 5 minutes of your time.

Your responses are confidential, and you don't need to answer any questions if you don't want to.

Date: ______/________/__________

1. What format did you use to complete the screening questionnaires?

☐ 1 In person ☐ 2 Phone ☐ 3 Online (via email link) ☐ 4 Returned by mail

2. Were the reasons & procedures for completing the screening questionnaires well-explained?

☐ 1 No ☐ 2 Yes

3. The screening questionnaires procedures were easy to complete.

☐ 1	☐ 2	☐ 3	☐ 4	☐ 5
**Strongly disagree**	**Disagree**	**Don't know**	**Agree**	**Strongly Agree**

4. The screening questionnaires procedures were easy to understand.

☐ 1	☐ 2	☐ 3	☐ 4	☐ 5
**Strongly disagree**	**Disagree**	**Don't know**	**Agree**	**Strongly Agree**

5. The time it took to complete the screening questionnaires was acceptable.

☐ 1	☐ 2	☐ 3	☐ 4	☐ 5
**Strongly disagree**	**Disagree**	**Don't know**	**Agree**	**Strongly Agree**

6. Which questionnaire best described your experience?

☐ 1 **Fear of Cancer Recurrence Inventory – Short Form (FCRI-SF)** (first questionnaire)

☐ 2 **Fear of Progression Questionnaire – Short Form (FoP-Q-SF)** (second questionnaire)

7. How important do you think it is to screen for fear of cancer returning or progressing? When do you think is the best time to screen for fear of cancer returning or progressing?

____

____

Please add any additional comments or feedback about the *Fear-Less* Program's screening questionnaires.

____

____

Thank you for participating in this survey.

THE PATIENT EXPERIENCE SURVEY

FEAR-LESS POST SELF-MANAGEMENT INTERVENTION

Thank you for agreeing to participate in this survey about the '*Fear-Less*' Program. You have been asked to complete this survey because you have completed the program's screening questionnaires, received the self-management intervention. When you see the term 'self-management intervention' we mean the booklet and the follow up phone calls you received.

This survey is about your experiences with the self-management intervention. The survey may take up to 10 minutes of your time. Your responses are confidential, and you are free to withdraw from the survey at any stage.

Date: _____/______/________

1. Prior to this program, has anyone ever discussed fear of cancer returning or progressing with you?

☐ 0 No

☐ 1 Yes

If yes, please tell us who discussed this with you and what happened next?
____

2. Prior to this program, have you previously received information, treatment or support to improve your fear of cancer returning or progressing?

☐ 0 No (proceed to Question 4)

☐ 1 Yes (please complete Q3a and 3b)

2a. Please specify the type of support you received for your fear:

☐ 0 Psychological treatment

☐ 1 Medical treatment

☐ 2 Professional advice. Explain ________________________________

☐ 3 Information or fact sheets

☐ 4 Other: ____

2b. Please specify who recommended this support

☐ 0 I sought it myself

☐ 1 My medical team (e.g., oncologist; doctors; nurses)

☐ 2 My friends or family

☐ 3 Other: ______________________________________

3. **What has stopped you from seeking help for your fear of cancer returning or progressing in the past?**

☐ 0 I did not have the time

☐ 1 I was embarrassed about seeking help

☐ 2 I did not know what to expect from the help or support

☐ 3 I was worried I would feel more upset or anxious

☐ 4 I felt that I had enough support from elsewhere

☐ 5 I thought that I did not need help

☐ 6 Other: ____

4. **Thinking about the '*Fear-Less*'Program self-management intervention you have just received, please rate the amount of the booklet that you have read:**

☐ 0 0% of the booklet (none) ☐ 1 25% of the booklet ☐ 2 50% of the booklet ☐ 3 75% of the booklet ☐ 4 100% of the booklet

5. **How many exercises in the booklet did you complete?** (i.e., Finding Your Values; Setting Goals; Relaxation Diary ; Getting To Know Your Triggers; Thought Diary; Looking at Your Control; Your Plan To Manage Fear)

☐ 0 0 ☐ 1 1 ☐ 2 2 ☐ 3 3 ☐ 4 4 ☐ 5 5 ☐ 6 6 ☐ 7 7

6. **Which of the exercises in the booklet were most helpful?** (You can tick more than one)

☐ 1 Finding Your Values

☐ 2 Setting Goals

☐ 3 Relaxation diary

☐ 4 Getting To Know Your Triggers

☐ 5 Thought Diary

☐ 6 Looking At Your Control

☐ 7 Your Plan To Manage Fear

☐ 6 Other (please specify) ____

7. **Thinking about the '*Fear-Less*'Program self-management intervention you have just received, what aspects of your care were <u>most</u> useful and helpful?**

____

____

____

8. **Thinking about the '*Fear-Less*' Program self-management intervention you have just received, what aspects of your care were <u>least</u> useful and helpful?**

____

____

____

9. Which of the following best describes any change in your fear of cancer returning or progressing since receiving the '*Fear-Less*' self-management intervention? (please tick)

☐ 1 My fear is much worse

☐ 2 My fear is somewhat worse

☐ 3 My fear has not changed

☐ 4 My fear is somewhat better

☐ 5 My fear is much better

10. To what extent do you think the changes in your fear are related to your self-management intervention?

☐ 1	☐ 2	☐ 3
**Not at all related**	**Partly related**	**Completely related**

11. What is your preferred way of receiving the *Fear-Less* follow-up care for fear of cancer returning or progressing? (Please tick)

☐ 1 Telephone

☐ 2 Video-conferencing

☐ 3 Face-to-face by myself (one on one with a clinician)

☐ 4 Face-to face in a group

☐ 5 Online

☐ 6 Paper based resource/information

12. Would you recommend the *Fear-Less* program to other patients with cancer?

☐ 1	☐ 2	☐ 3
**Yes**	**Don't know**	**No**

13. What are your suggestions about how we can improve the '*Fear-Less'* Program or the intervention you received?

____

____

____

____

Thank you for participating in the survey.

The Patient Experience Survey

Fear-Less Post *Conquer Fear* Intervention

Thank you for agreeing to participate in this survey about the '*Fear-Less*' Program. You have been asked to complete this survey because you have completed the program's screening questionnaires, received a referral for the *Conquer Fear* psychology intervention and have completed that follow-up care.

This survey is about your experiences with the *Conquer Fear* psychology intervention. The survey may take up to 10 minutes of your time. Your responses are confidential, and you are free to withdraw from the survey at any stage.

Date: _____/______/________

1. Prior to this program, has anyone ever discussed fear of cancer returning or progressing with you?

☐ 0 No

☐ 1 Yes

If yes, please tell us who discussed this with you and what happened next?
____

2. Prior to this program, have you previously received information, treatment or support to improve your fear of cancer returning or progressing?

☐ 0 No (proceed to Question 4)

☐ 1 Yes (please complete Q3a and 3b)

2a. Please specify the type of support you received for your fear:

☐ 0 Psychological treatment

☐ 1 Medical treatment

☐ 2 Professional advice. Explain _______________________________

☐ 3 Information or fact sheets

☐ 4 Other: ____

2b. Please specify who recommended this support

☐ 0 I sought it myself

☐ 1 My medical team (e.g., oncologist; doctors; nurses)

☐ 2 My friends or family

☐ 3 Other: ____________________________________

3. **What has stopped you from seeking help for your fear of cancer returning or progressing in the past?**

☐ 0 I did not have the time

☐ 1 I was embarrassed about seeking help

☐ 2 I did not know what to expect from the help or support

☐ 3 I was worried I would feel more upset or anxious

☐ 4 I felt that I had enough support from elsewhere

☐ 5 I thought that I did not need help

☐ 6 Other: ____

4. **Thinking about the '*Fear-Less'* Program *Conquer Fear* psychology intervention you have just received, what aspects of your care were <u>most</u> useful and helpful?**

____

____

____

5. **Thinking about the '*Fear-Less'* Program *Conquer Fear* psychology intervention you have just received, what aspects of your care were <u>least</u> useful and helpful?**

____

____

____

6. **Which of the following best describes any change in your fear of cancer returning or progressing since receiving your *Conquer Fear* psychology intervention in the '*Fear-Less*' Program?** (please tick)

☐ 1 My fear is much worse

☐ 2 My fear is somewhat worse

☐ 3 My fear has not changed

☐ 4 My fear is somewhat better

☐ 5 My fear is much better

7. **To what extent do you think the changes in your fear are related to your *Conquer Fear* psychology intervention?**

☐ 1	☐ 2	☐ 3
**Not at all related**	**Partly related**	**Completely related**

8. What is your preferred way of receiving the *Conquer Fear* psychology intervention for fear of cancer returning or progressing? (Please tick)

☐1 Telephone

☐2 Video-conferencing

☐3 Face-to-face by myself (one on one with a clinician)

☐4 Face-to face in a group

☐5 Online

☐6 Paper based resource/information

9. Would you recommend the *Fear-Less* program to other patients with cancer?

☐1	☐2	☐3
**Yes**	**Don't know**	**No**

10. What are your suggestions about how we can improve the '*Fear-Less'* Program or the psychology intervention you received?

____

____

____

____

Thank you for participating in the survey.
